# Uso combinado de marcadores sorológicos e análise espacial na vigilância epidemiológica da hanseníase

**DOI:** 10.26633/RPSP.2021.129

**Published:** 2021-11-19

**Authors:** Gabriela de Cássia Ribeiro, Josafá Gonçalves Barreto, Isabela de Caux Bueno, Bruna Oliveira Costa, Francisco Carlos Félix Lana

**Affiliations:** 1 Universidade Federal dos Vales do Jequitinhonha e Mucuri (UFVJM) Departamento de Enfermagem Diamantina (MG) Brasil Universidade Federal dos Vales do Jequitinhonha e Mucuri (UFVJM), Departamento de Enfermagem, Diamantina (MG), Brasil.; 2 Universidade Federal do Pará (UFPA) Laboratório de Epidemiologia Espacial (LabEE) Castanhal (PA) Brasil Universidade Federal do Pará (UFPA), Laboratório de Epidemiologia Espacial (LabEE), Castanhal (PA), Brasil.; 3 Universidade Federal de Minas Gerais (UFMG) Departamento de Saúde Materno-Infantil Belo Horizonte (MG) Brasil Universidade Federal de Minas Gerais (UFMG), Departamento de Saúde Materno-Infantil, Belo Horizonte (MG), Brasil.; 4 Universidade Federal dos Vales do Jequitinhonha e Mucuri (UFVJM) Programa Multicêntrico de Pós-graduação em Ciências Fisiológicas Diamantina (MG) Brasil Universidade Federal dos Vales do Jequitinhonha e Mucuri (UFVJM), Programa Multicêntrico de Pós-graduação em Ciências Fisiológicas, Diamantina (MG), Brasil.

**Keywords:** Hanseníase, *Mycobacterium leprae*, monitoramento epidemiológico, sorologia, análise espacial, Brasil, Leprosy, *Mycobacterium leprae*, epidemiological monitoring, serology, spatial analysis, Brazil, Lepra, *Mycobacterium leprae*, monitoreo epidemiológico, serología, análisis espacial, Brasil

## Abstract

**Objetivo.:**

Avaliar o uso combinado de marcadores sorológicos e análise espacial para ampliar a sensibilidade da vigilância epidemiológica da hanseníase.

**Método.:**

Este estudo transversal foi realizado com vizinhos de casos de hanseníase e familiares e vizinhos de escolares com sorologia positiva anti-glicolipídeo fenólico I (PGL-I) em Diamantina, Minas Gerais, Brasil. Definiram-se como vizinhos as pessoas que residiam em um raio de até 100 metros de escolares e de casos de hanseníase. Para a coleta de dados, foram realizados entrevista semiestruturada, exame dermatoneurológico e teste sorológico rápido ML Flow. Todos os endereços foram georreferenciados. Foram realizadas regressão multivariada e análise espacial, tendo a sororreatividade anti-PGL-I como variável dependente.

**Resultados.:**

Foram estudadas 1 491 pessoas: 1 009 (67,7%) familiares e vizinhos dos escolares com sorologia positiva e 482 (32,3%) vizinhos dos casos de hanseníase. Do total, 421 (28,2%) apresentaram soropositividade anti-PGL-I. A chance de soropositividade foi maior entre familiares e vizinhos dos escolares soropositivos (*P* < 0,001), entre pessoas com renda familiar de 1 salário-mínimo (*P* < 0,001), entre os mais jovens (*P* < 0,001) e entre os que residiam em domicílios com um a cinco cômodos (*P* = 0,007). A taxa de soropositividade foi maior em área geográfica correspondente aos escolares soropositivos (*P* < 0,001), ou seja, houve divergência entre o foco de maior concentração de casos e o de maior soropositividade.

**Conclusões.:**

O uso combinado de marcadores sorológicos e análise espacial possibilitou identificar fragilidades operacionais dos serviços e uma possível endemia oculta de hanseníase nos setores censitários urbanos do município. Atividades de rastreamento de contatos sociais e vizinhos, busca ativa, campanhas educativas, inquéritos escolares e análise do território facilitam o diagnóstico precoce da hanseníase.

## Abstract (en)

**Objective.:**

To evaluate the combined use of serologic markers and spatial analysis to increase the sensitivity of leprosy epidemiological surveillance.

**Method.:**

This cross-sectional study was performed with neighbors of leprosy cases and neighbors and family members of schoolchildren with a positive anti-phenolic glycolipid I (PGL-I) test in Diamantina, state of Minas Gerais, Brazil. Neighbors were those living within a 100-m radius of schoolchildren or leprosy cases. Data collection included a semi-structured interview, dermatologic examination, and rapid ML Flow test. All residential addresses were georeferenced. Multivariate regression and spatial analysis were performed with anti-PGL-I seropositivity as the dependent variable.

**Results.:**

The study included 1 491 individuals: 1 009 (67.7%) family members and neighbors of schoolchildren with positive serology test and 482 (32.3%) neighbors of leprosy cases. Of the total, 421 (28.2%) were positive for anti-PGL-I. Seropositivity was higher among family members and neighbors of seropositive schoolers (*P* < 0.001), among people with family income of 1 minimum wage (*P* < 0.001), among the youngest participants (*P* < 0.001), and among those living in homes with one to five rooms (*P* = 0.007). The seropositivity rate was higher in the geographic area corresponding to seropositive schoolchildren (*P* < 0.001), that is, the spot with the highest number of cases was different from the spot with the highest rate of seropositivity.

**Conclusions.:**

The combined use of serologic markers and spatial analysis allowed us to easily identify operational weaknesses of services and a possible occult leprosy endemism in the municipality’s urban census tracts. Tracing of social contact and neighbors, active search, educational campaigns, school surveys, and territorial analyses facilitate the early diagnosis of leprosy.

## RESUMEN

**Objetivo.:**

Evaluar el uso conjunto de los marcadores serológicos y del análisis espacial para ampliar la sensibilidad de la vigilancia epidemiológica de la lepra.

**Método.:**

Este estudio transversal se realizó con vecinos de personas con casos de lepra y con familiares y vecinos de escolares con resultados positivos en las pruebas serológicas del antígeno glicolípido fenólico I (PGL-I) en Diamantina (Minas Gerais, Brasil). Se definieron como vecinos las personas residentes en un radio de hasta 100 metros de distancia de los escolares y de las personas con casos de lepra. Para la recopilación de datos se realizaron entrevistas semiestructuradas, exámenes dermatoneurológicos y pruebas serológicas rápidas ML Flow. Todas las direcciones se ubicaron por georreferencia. Se realizó una regresión multivariante y un análisis espacial, y se empleó la reactividad serológica anti-PGL-I como variable dependiente.

**Resultados.:**

Se estudiaron 1 491 personas: 1 009 (67,7%) familiares y vecinos de escolares con resultados positivos en las pruebas serológicas y 482 (32,3%) vecinos de las personas con casos de lepra. Del total, 421 (28,2%) presentaron seropositividad anti-PGL-I. La posibilidad de presentar seropositividad fue mayor en los familiares y vecinos de los escolares seropositivos (*P* < 0,001), en las personas con ingresos familiares de un salario mínimo (*P* < 0,001), en las personas más jóvenes (*P* < 0,001) y en los residentes en domicilios con una a cinco habitaciones (*P* = 0,007). La tasa de seropositividad fue mayor en la zona geográfica correspondiente a los escolares seropositivos (*P* < 0,001), por lo que hubo divergencia entre el foco de mayor concentración de casos y el de mayor seropositividad.

**Conclusiones.:**

El uso conjunto de los marcadores serológicos y del análisis espacial permitió determinar las fragilidades operativas de los servicios y una posible endemia oculta de lepra en los sectores urbanos del municipio incluidos en el censo correspondiente. Las actividades de rastreo de contactos sociales y cercanos, la búsqueda activa de casos, las campañas educativas, las encuestas escolares y el análisis del territorio facilitan el diagnóstico temprano de la lepra.

A hanseníase, considerada uma doença tropical negligenciada, está estreitamente relacionada à desigualdade social e à dificuldade de acesso aos serviços básicos de saúde pela população acometida (1). Em 2019, 29 936 casos novos de hanseníase foram registrados em 26 países da região das Américas. No Brasil, responsável por 93,1% desses diagnósticos (2), a existência de uma Diretriz Nacional para padronizar a vigilância e a atenção à hanseníase não foi capaz por si só de controlar a doença, que ainda se mantém como um problema de saúde pública no país (3).

O cenário epidemiológico da hanseníase no Brasil apresenta grande heterogeneidade, com as maiores taxas de prevalência registradas nos estados das regiões Centro-Oeste e Norte (4). Ao mesmo tempo, mesmo em regiões de menor endemicidade, os municípios apresentam, de modo geral, alta taxa de detecção de casos novos tanto para a população em geral quanto para menores de 15 anos, inclusive com diagnósticos de incapacidades físicas (5). As altas taxas revelam a existência de uma prevalência oculta de casos da doença (6, 7).

O diagnóstico da hanseníase, essencialmente clínico-epidemiológico, é um grande desafio para os profissionais de saúde, pois muitos não possuem capacitação técnica para identificar os principais sinais e sintomas da doença em sua fase inicial (4). Ademais, os exames disponíveis, principalmente a baciloscopia, contribuem para a confirmação diagnóstica e classificação operacional, mas não descartam a presença da doença diante de um resultado negativo (3).

Por sua vez, a vigilância epidemiológica da hanseníase tem como principais objetivos a detecção e o tratamento precoce dos casos novos; a descentralização das ações de detecção e controle, de modo a integrá-las na atenção primária à saúde (APS); e a vigilância de contatos domiciliares, familiares e sociais (3). Deve estar presente em toda a rede de atenção à saúde (RAS), a fim de subsidiar informações sobre distribuição espacial, magnitude e carga da hanseníase nas diferentes regiões do país (3).

Entretanto, especialistas afirmam a necessidade de estabelecer estratégias inovadoras que incrementem a vigilância epidemiológica da hanseníase, como controle rigoroso de contatos, identificação da infecção subclínica e mapeamento das áreas de risco (8). A Estratégia Nacional para Enfrentamento da Hanseníase 2019-2022, elaborada pelo Ministério da Saúde, segue essa perspectiva ao enfatizar a importância de se reconhecer a epidemiologia e a dinâmica da hanseníase no território. Ainda, inclui, no escopo das ações de controle, todos os municípios brasileiros, classificando-os por categorias de acordo com o nível de endemia e necessidades específicas (4).

Nesse cenário, os testes sorológicos e as técnicas de epidemiologia espacial surgem como alternativas para o fortalecimento da vigilância da hanseníase. Os testes sorológicos auxiliam no rastreamento de pessoas com risco de desenvolverem hanseníase entre os contatos de casos (9) e, entre os indivíduos saudáveis, possibilitam a descoberta de uma cadeia de transmissão ativa (10). Consistem na detecção de anticorpos, principalmente da classe IgM, contra antígenos específicos do *Mycobacterium leprae* (*M. leprae*) (11). O antígeno padronizado e estabelecido como o melhor marcador de infecção pelo *M. leprae* é o glicolipídeo fenólico I (PGL-I), em suas formas nativa e semissintética, pois se mostra mais específico em relação aos outros (12).

A utilização das técnicas de análise espacial pode ajudar a revelar o padrão de transmissão da hanseníase ou da infecção pelo *M. leprae* (13). Assim, pode contribuir para o planejamento das ações de controle de acordo com as especificidades de cada grupo populacional, possibilitando aos gestores alocar recursos para as áreas de maior transmissão e risco de adoecimento (14, 15).

Nesse sentido, o objetivo do presente estudo foi avaliar o uso combinado de marcadores sorológicos e da análise espacial para a ampliação da sensibilidade das ações de vigilância epidemiológica da hanseníase.

## MATERIAIS E MÉTODOS

O presente estudo, do tipo transversal, descritivo e analítico, é derivado de um estudo maior, denominado “Prevalência e distribuição da infecção pelo *M. leprae* por meio de marcadores sorológicos e geoprocessamento em Diamantina, Minas Gerais” (16). Foi realizado nos setores censitários urbanos de Diamantina, município situado no Vale do Jequitinhonha, em Minas Gerais, com estimativa populacional de 47 825 habitantes para o ano de 2020. O município se divide em 69 setores censitários urbanos e rurais (17). De 2010 a 2019, o município apresentou uma taxa média de detecção de casos novos de hanseníase de 4,02/100 000 habitantes (5), o que o classifica como de média endemicidade de acordo com os parâmetros do Ministério da Saúde (endemicidade baixa: < 2,00/100 000 habitantes; média: 2,00 a 9,99/100 000; alta: 10,00 a 19,99/100 000; muito alta: 20,00 a 39,99/100 000; hiperendemicidade: > 40,0/100 000 habitantes) (3).

Como público-alvo para cada fase de coleta de dados, foram selecionados: 1) casos de hanseníase e seus contatos; 2) escolares de 7 a 14 anos do município; e 3) familiares e vizinhos dos escolares soropositivos e vizinhos de casos de hanseníase. Foram incluídos familiares que residiam no mesmo domicílio e vizinhos de 32 escolares que apresentaram sorologia positiva anti-PGL-I na fase anterior da pesquisa (6), bem como vizinhos de 13 casos de hanseníase multibacilar que moravam há mais de 5 anos no mesmo domicílio. Considerou-se como vizinho todo indivíduo que residia em um raio de até 100 metros de distância da residência dos escolares soropositivos e dos casos de hanseníase, com mais de 5 anos de idade e sem histórico de hanseníase.

Inicialmente, todos os participantes ou seus responsáveis foram convidados a assinar um termo de consentimento livre e esclarecido específico por faixa etária. Como garantia de anonimato, foram asseguradas aos participantes a não divulgação dos nomes e a utilização dos dados apenas para fins científicos. A equipe de pesquisadores foi composta por enfermeiros treinados pela pesquisadora responsável. Houve apoio de uma dermatologista e de uma enfermeira, ambas com experiência em hanseníase, para encaminhamento de casos sugestivos e discussão de possíveis sinais e sintomas.

A coleta de dados ocorreu nos meses de maio a novembro de 2018. As entrevistas semiestruturadas abordaram aspectos socioeconômicos e demográficos como renda, cor, escolaridade, condições e tempo de moradia e de convivência com caso de hanseníase ou escolar com sorologia anti-PGL-I positiva e conhecimentos sobre hanseníase. A fim de detectar possíveis sinais e sintomas de hanseníase, foi realizado exame dermatoneurológico de acordo com as diretrizes do Ministério da Saúde (3).

Para o exame sorológico, foi realizada punção digital e utilizado o teste rápido ML Flow do lote ML2018/01, produzido pelo Laboratório de Desenvolvimento e Produção de Testes Rápidos do Instituto de Patologia Tropical e Saúde Pública da Universidade Federal de Goiás (LDPTR/IPTSP/UFG). O ML Flow é um teste imunológico que detecta com alta especificidade anticorpos IgM anti-PGL-I, proteína específica do *M. leprae*, em amostras de sangue total ou soro. Se o anticorpo for específico, ele se ligará ao antígeno e uma linha vermelha aparecerá na zona de teste. Caso contrário, apenas a linha controle aparecerá positiva. O teste possui uma classificação qualitativa (negativo ou positivo) e quantitativa (graduação de 1+ a 4+), de acordo com a intensidade de coloração na linha do teste (18). Foram utilizados 5 µl de sangue total no suporte de papel do receptáculo de amostras e, posteriormente, foram adicionadas duas gotas de reagente ao receptáculo. A leitura visual foi feita 5 minutos após a realização do teste, conforme as orientações do fabricante.

Os testes sorológicos, principalmente aqueles realizados pela técnica de ELISA, são considerados mais sensíveis do que a baciloscopia e a biópsia de pele. Entretanto, possuem baixa sensibilidade para os pacientes paucibacilares e baixa especificidade na população saudável, pois muitos soropositivos não desenvolverão a doença (19). O teste ML Flow é indicado em pesquisas de campo desde a década de 2000 por demonstrar alta concordância com o ELISA (91,0%), mas possuir menor custo e maior facilidade de coleta, leitura e interpretação dos dados (18).

Um banco de dados foi elaborado no *software* Epi Info, versão 3.5.1. Após dupla digitação e correção de inconsistências, os dados foram exportados para o programa *Statistical Package for the Social Sciences* (SPSS), versão 25, para tratamento e análise. A associação entre as variáveis independentes (socioeconômicas, demográficas, condições de moradia e de convivência com o caso ou o escolar soropositivo e conhecimentos sobre hanseníase) e a variável dependente (sororreatividade anti-PGL-I) foi avaliada por análise univariada através dos testes do qui-quadrado de Pearson ou exato de Fisher, quando pertinente. O nível de significância adotado foi de *P* < 0,05.

Para o método *forward* (critério de entrada das variáveis), foi realizada análise univariada através do ajuste dos respectivos modelos de regressão para cada variável. Nessa etapa, permaneceram as variáveis com nível de significância de até 25% (*P* = 0,25) ou que se mostraram importantes de acordo com a literatura. As variáveis selecionadas foram inseridas em uma regressão multivariada. O *software* utilizado nas análises foi o R, versão 3.5.0.

Considerando a dependência espacial entre bairros, com o objetivo de verificar a influência das variáveis sobre o resultado do teste ML Flow, utilizou-se o modelo de equações de estimativas generalizadas (*generalized estimating equations*) para quantificar a correlação existente entre medidas repetidas. Todos os endereços foram georreferenciados por meio de técnicas de geoprocessamento utilizando o *software* livre QGIS, versão 2.18.0, e imagem de satélite georreferenciada que mostra toda a mancha urbana da cidade de Diamantina. O mapa de setores censitários do município foi utilizado como a menor unidade espacial de agregação dos dados (15).

Para a coleta de dados da população-alvo, uma camada *shape file* contendo as localizações dos escolares com sorologia anti-PGL-I positiva e de casos de hanseníase foi importada para o aplicativo Mapit GIS, versão 6.5.0 (Mapit GIS LTD, Reino Unido), possibilitando localizar e georreferenciar os endereços constantes dentro da área de interesse, no caso, o *buffer* de 100 metros. As análises espaciais dessa etapa foram realizadas no *software* gratuito QGIS, versão 3.4 Madeira (www.qgis.org).

A investigação foi aprovada pelo Comitê de Ética em Pesquisa da Universidade Federal de Minas Gerais (UFMG), em Belo Horizonte (CAAE 54556716.5.0000.5149).

## RESULTADOS

Foram incluídas no estudo 1 491 pessoas, das quais 1 009 (67,7%) eram familiares e vizinhos dos escolares com sorologia positiva anti-PGL-I, e 482 (32,3%) eram vizinhos dos casos de hanseníase. Do total, 28,2% (n = 421) apresentaram soropositividade anti-PGL-I. A mediana da idade dos participantes foi de 37 anos. A renda familiar média foi de 1,66 salário-mínimo, e 99,5% não apresentavam manchas ou outros sinais e sintomas sugestivos de hanseníase.

A mediana (± desvio padrão) de tempo de moradia no domicílio para essa população foi de 15 anos, com mínimo de 1 e máximo de 88 anos. As residências possuíam, em média, 6,4 cômodos (± 1,9), 2,7 quartos (± 1,0) e 4,0 moradores (± 1,9). A proporção de participantes que relataram dividir o quarto com ao menos mais uma pessoa foi de 68,2% (± 0,7).

A tabela 1 contempla as variáveis explicativas do desfecho prevalência de soropositividade anti-PGL-I na população de estudo. A maior chance de apresentar soropositividade anti-PGL-I foi entre os familiares ou vizinhos dos escolares soropositivos (*odds ratio* [OR]: 3,64; intervalo de confiança de 95% [IC95%]: 2,47; 4,44, *P* < 0,001); entre os que recebiam 1 salário-mínimo (US$ 251,00 em 2018) ou mais (OR: 2,05; IC95%: 1,47; 2,44, *P* < 0,001); entre os que tinham menos de 15 anos (OR: 1,89; IC95%: 1,33; 2,25, *P* < 0,001); e entre os que residiam em domicílio com um a cinco cômodos (OR: 1,44; IC95%: 1,10; 1,65, *P* = 0,007).

**FIGURA 1.**
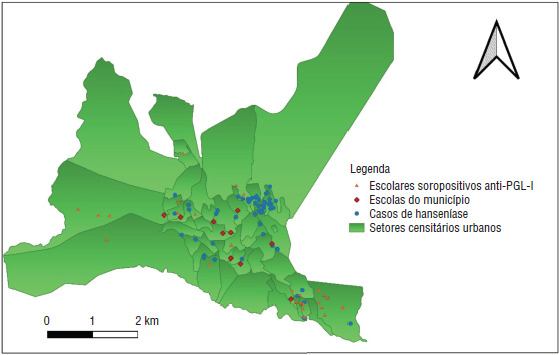
Distribuição espacial de 42 casos de hanseníase e 33 escolares com soropositividade anti-PGL-I nos setores censitários urbanos, Diamantina (MG), Brasil, 2018^a^

^a^ PGL-I: glicolipídeo fenólico I.

A figura 1 mostra a distribuição espacial dos casos de hanseníase e dos escolares com sorologia positiva anti-PGL-I nos setores censitários urbanos. É possível perceber uma diferença entre as áreas de concentração das duas categorias populacionais, isto é, casos de hanseníase e escolares soropositivos.

Em relação à população total do estudo, 24,8% residiam no bairro Rio Grande (n = 369), no qual houve maior concentração de casos da doença. O bairro da Palha, onde residiam 24,4% dos entrevistados (n = 364), teve o maior número de indivíduos soropositivos para anti-PGL-I. Os 50,8% restantes estavam distribuídos nos demais bairros da zona urbana do município.

**FIGURA 2.**
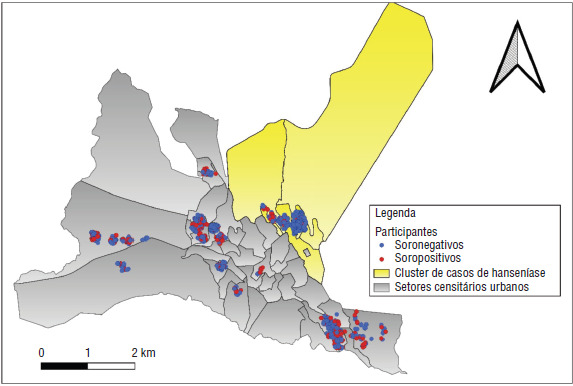
Distribuição espacial de 1 491 indivíduos entrevistados dentro e fora do *cluster* de adoecimento, Diamantina (MG), Brasil, 2018

Verifica-se, na figura 2, a existência de um *cluster* de casos de hanseníase, detectado em fase anterior ao estudo (6). Do total de soropositivos (n = 421), 353 (83,8%) residiam fora do *cluster* de casos, demonstrando, com significância estatística (*P* < 0,001), maior soropositividade nos setores censitários que correspondem ao bairro Palha, local com o maior número de escolares com sorologia anti-PGL-I positiva.

**FIGURA 3.**
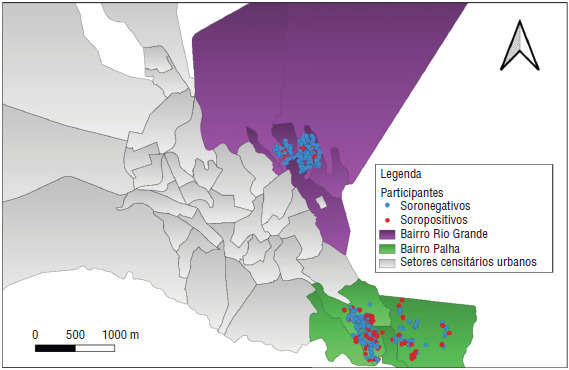
Comparação da soropositividade anti-PGL-I entre os setores censitários dos bairros Palha e Rio Grande, Diamantina (MG), Brasil, 2018

Complementarmente, a figura 3 demonstra os setores censitários dos bairros com maior soropositividade anti-PGL-I. O bairro Palha apresentou 21,8% (n = 92) de pessoas soropositivas e corresponde à área de maior número de escolares com sorologia anti-PGL-I. O bairro Rio Grande apresentou 16,2% (n = 68) de soropositividade e é o *cluster* de casos de hanseníase. Ao compará-los, foi identificada, com significância estatística, maior prevalência de soropositividade no bairro Palha (*P* = 0,002).

## DISCUSSÃO

Esta investigação identificou alta taxa de soropositividade anti-PGL-I na população de estudo, composta por pessoas saudáveis e sem sinais e sintomas de hanseníase. De fato, observaram-se, no município de Diamantina, características epidemiológicas que indicavam diagnósticos tardios e falhas operacionais nas ações de controle da doença (20). Houve variação na taxa de detecção de hanseníase ao longo de uma série histórica, de média a alta, com períodos silenciosos (5). Além disso, um inquérito realizado na região demonstrou que 73,2% dos casos foram diagnosticados com as formas bacilíferas e que 78,1% dos casos apresentavam algum grau de incapacidade física (20).

**TABELA 1.** Regressão marginal multivariada para determinar variáveis explicativas da prevalência de soropositividade anti-PGL-I em 1 491 participantes, Diamantina (MG), Brasil, 2018

Variáveis	Modelo inicial^a^			Modelo final^a^		
	OR	IC95%	*P*	OR	IC95%	*P*
Relação caso	1			1		
Relação escolar	3,54	2,38; 5,26	< 0,001^b^	3,64	2,47; 4,44	< 0,001^b^
Anos de estudo						
> 9 anos	1					
5 a 9 anos	1,08	0,76; 1,54	0,656			
1 a 4 anos	0,88	0,67; 1,17	0,387			
Sem escolaridade	1,41	0,96; 2,07	0,08			
Renda						
< 1 salário-mínimo	1			1		
≥ 1 salário-mínimo	2,19	1,57; 3,04	< 0,001^b^	2,06	1,47; 2,44	< 0,001^b^
Faixa etária						
15 a 40 anos	1			1		
< 15 anos	2,23	1,50; 3,33	< 0,001^b^	1,89	1,33; 2,25	< 0,001^b^
> 40 anos	1,31	0,94; 1,82	0,111	1,3	0,96; 1,52	0,09
Cor						
Parda	1					
Branca	1,23	0,89; 1,68	0,206			
Preta	1,21	0,90; 1,63	0,204			
Tempo de residência no endereço						
> 15 anos	1					
1 a 15 anos	1,03	0,80; 1,33	0,826			
Número de cômodos em casa						
≥ 6	1			1		
1 a 5	1,34	0,97; 1,84	0,074	1,44	1,10; 1,65	0,007^b^
Número de quartos em casa						
≥ 3	1					
< 3	1,13	0,83; 1,53	0,45			
Conhece alguém que já teve hanseníase						
Sim	1					
Não	1,49	0,99; 2,22	0,053			

a OR: *odds ratio*; IC95%: intervalo de confiança de 95%; *P*: associação significativa conforme teste do qui-quadrado (*P* < 0,05).

b Significância estatística.

Outros estudos brasileiros realizados em áreas classificadas como não endêmicas relataram resultados semelhantes. Durante uma campanha realizada com pessoas saudáveis em Jardinópolis, no estado de São Paulo, município onde o número de habitantes é semelhante ao do cenário deste estudo, foi detectada uma taxa de 23,3% de soropositividade anti-PGL-I (21). No Distrito Federal, considerado não endêmico desde 2005, foi identificada soropositividade anti-PGL-I de 34,3% em indivíduos que desconheciam contato com a hanseníase. Do total de 434 examinados, 10,1% (n = 44) foram diagnosticados com a doença; desses 44, 88,6% foram classificados como multibacilares (10). Esses achados, juntamente com os resultados do presente estudo, corroboram categoricamente o novo entendimento da Estratégia Nacional para Enfrentamento da Hanseníase 2019-2022 (4) e do Plano Estadual de Enfrentamento da Hanseníase em Minas Gerais (22), na medida que demonstram a importância de se discutir a hanseníase e a cadeia de transmissão ativa nos municípios de forma individual, independentemente dos parâmetros de endemicidade.

Segundo o novo plano proposto pelo estado de Minas Gerais, Diamantina se enquadra no grupo 3, o qual é composto por municípios que apresentaram uma taxa de detecção ≥ 10 casos/100 000 habitantes no período de 2013 a 2017. Entre as diversas estratégias elencadas para esse grupo, destacam-se duas consideradas um avanço nas discussões sobre o uso de novas ferramentas na vigilância epidemiológica da hanseníase: a utilização dos sistemas de informações geográficas para análise da situação da hanseníase no território e a inserção das sorologias no rol dos exames presentes nas capacitações para os profissionais da rede de laboratório (22).

Em relação ao presente estudo, dadas as particularidades observadas, infere-se que a endemia seja local. Apesar de Diamantina ser uma cidade universitária, as características relacionadas à idade, à escolaridade e ao tempo médio de moradia não correspondem ao público flutuante.

A soropositividade anti-PGL-I foi mais evidente entre os que residiam em domicílios com menos cômodos. Apesar de as moradias das pessoas afetadas pela hanseníase no Brasil serem maiores do que aquelas identificadas na Índia e na Indonésia (23), ainda não são consideradas ideais para o tamanho das famílias, principalmente porque a maior parte desses indivíduos vivem em situação de vulnerabilidade social (24).

A influência da situação socioeconômica sobre a ocorrência da soropositividade anti-PGL-I também foi evidenciada em relação à renda familiar, que se apresentou em torno de 1 salário-mínimo. A hanseníase, além de atingir mais frequentemente as pessoas em idade economicamente ativa, pode gerar incapacidades físicas, contribuindo para a manutenção da relação da doença com piores condições de vida e reforçando o seu caráter de doença negligenciada (1, 24). Há evidências de que os programas de transferência de renda interferem positivamente na diminuição das taxas de detecção da hanseníase, pois promovem redução da desigualdade social e maior desenvolvimento humano (25).

A prevalência de soropositividade anti-PGL-I entre a população mais jovem, apesar da ausência de registros de notificações de hanseníase em menores de 15 anos, chama a atenção para uma cadeia de transmissão local e ativa da hanseníase. Diante do longo período de incubação da doença, a soropositividade em crianças e adolescentes indica uma infecção precoce (26), aumentando a chance de esses jovens se tornarem adultos doentes e com incapacidades físicas (27, 28), uma vez que diagnósticos em crianças ainda são um desafio para os profissionais de saúde (29). Sendo assim, verifica-se também a necessidade de ampliar as estratégias de vigilância para essa população, incluindo creches e escolas, tendo a sorologia anti-PGL-I se mostrado um importante marcador de monitoramento epidemiológico (29, 30).

Deve-se destacar a importância dos escolares neste estudo, os quais serviram como ponto de partida para o inquérito com a população-alvo. A elevada prevalência de soropositividade em torno deles aponta para uma infecção extradomiciliar de casos de hanseníase (31) e para a importância da realização de inquéritos em grande escala no ambiente escolar, principalmente em regiões de maior vulnerabilidade social (8, 29, 32).

Os achados referentes à análise espacial vão ao encontro do cenário clínico-epidemiológico que se revelou por meio da soropositividade anti-PGL-I. Apesar de ter sido descrito anteriormente no município de estudo um *cluster* de adoecimento com relação espaço-temporal (6), a maior parte dos indivíduos saudáveis soropositivos se encontrava em setores censitários correspondentes ao bairro onde estava localizada a maioria dos escolares com sorologia anti-PGL-I positiva.

Essa configuração espacial, na qual há uma divergência entre o foco de maior concentração de casos da doença e o de maior soropositividade, prediz a existência de infecção ativa pelo *M. leprae* e uma prevalência oculta de casos novos da hanseníase (33). Estudos confirmam a existência de risco entre contatos sociais (escolares, de trabalho, religiosos, de lazer) e vizinhos (29, 34) e sugerem a necessidade de ampliação da rede de rastreamento de contatos, além da vigilância clínica dos indivíduos soropositivos (10, 29).

A ocorrência de soropositividade em todos os setores censitários incluídos no estudo, juntamente com uma área de maior soropositividade anti-PGL-I, reforça a reflexão sobre as fontes de infecção da hanseníase para além dos domicílios (34). Observaram-se, durante as visitas domiciliares, residências muito próximas e áreas de maior densidade populacional, fatores que podem ser determinantes para a manutenção da cadeia de transmissão do bacilo.

No município, tanto a área de concentração de casos quanto a de soropositividade são periféricas e com alta vulnerabilidade socioeconômica. Entretanto, a diferença observada entre elas foi que o *cluster* de adoecimento se localizou em setores censitários munidos de duas unidades de Estratégia Saúde da Família (ESF), com pelo menos uma equipe capacitada para o diagnóstico precoce da hanseníase. Em contrapartida, a área de maior soropositividade possuía somente uma ESF, com apenas um profissional treinado e com microáreas sem agentes comunitários de saúde. Sabe-se que o aumento da detecção de casos de hanseníase pode estar relacionado a uma melhor assistência à saúde (13). Sendo assim, a organização da vigilância em saúde, maior cobertura da ESF e equipe sensibilizada para uma atenção qualificada e equânime são fatores fundamentais para o diagnóstico precoce da hanseníase e para a ampliação das taxas de cura e contatos examinados (8, 33).

É consenso na literatura a existência de associação entre soropositividade anti-PGL-I e maior risco da ocorrência de casos de hanseníase. No entanto, não se pode afirmar que os testes sorológicos estimem a real prevalência da infecção pelo *M. leprae* na população devido à complexidade que envolve o sistema imunológico do indivíduo. Cerca de 60% dos doentes no polo tuberculoide não são reagentes à sorologia, e 90% dos infectados nunca desenvolverão a doença (9, 21). Ainda assim, este estudo trouxe evidências imunológicas da presença de uma cadeia de transmissão ativa e, possivelmente, de uma endemia oculta de hanseníase no município, o que justifica as características clínico-epidemiológicas encontradas em estudos anteriores (6, 20).

As limitações deste estudo incluem a impossibilidade de investigação em todos os setores censitários do município, a ausência de diagnósticos de casos de hanseníase e os limites inerentes aos testes sorológicos, por exemplo, a baixa sensibilidade em populações saudáveis. Entretanto, o estudo evidencia que ações de vigilância epidemiológica devem ser priorizadas para o sucesso dos programas de hanseníase, principalmente a busca ativa de casos. Essa estratégia apresenta potencial para ser utilizada como medida de saúde pública no Brasil e em outros países das Américas, pois, mesmo não considerados endêmicos, necessitam de vigilância frequente. Em 2019, alguns países registraram altas porcentagens de diagnósticos de hanseníase com grau 2 de incapacidade física, com destaque para Uruguai (35,3%), Peru (12,5%), Colômbia (12,7%) e Costa Rica (50,0%) (2).

Considera-se que o uso combinado dos testes sorológicos e da análise espacial cumpriu o papel de marcador epidemiológico no cenário deste estudo, uma vez que a sorologia positiva e a representação espacial promoveram sensivelmente a identificação de fragilidades operacionais, principalmente dos serviços da APS, e locais prioritários para intervenções dentro dos setores censitários urbanos do município.

Sabe-se que os desafios para cumprir as ações de vigilância epidemiológica da hanseníase são de ordens diversas —socioeconômicos, políticos, administrativos e técnicos. No entanto, reitera-se a importância da ampliação das estratégias de monitoramento em distintos espaços geográficos. Acredita-se que o rastreamento de contatos sociais e vizinhos, a intensificação da busca ativa e a realização de campanhas educativas, inquéritos escolares e análise do território contribuam fortemente para o diagnóstico precoce da hanseníase e consequente redução das incapacidades físicas.

## Declaração.

As opiniões expressas no manuscrito são de responsabilidade exclusiva dos autores e não refletem necessariamente a opinião ou política da RPSP/PAJPH ou da Organização Pan-Americana da Saúde (OPAS).
